# Small fiber polyneuropathy: A new therapeutic target in patients with interstitial cystitis/bladder pain syndrome?

**DOI:** 10.3389/fruro.2023.1098294

**Published:** 2023-02-16

**Authors:** Wyatt Whitman, Maxwell Sandberg, Peyton Lee, Stephen J. Walker

**Affiliations:** ^1^ Department of Urology, Atrium Health Wake Forest Baptist, Winston Salem, NC, United States; ^2^ Wake Forest School of Medicine, Institute for Regenerative Medicine, Winston Salem, NC, United States

**Keywords:** interstitial cystitis/bladder pain syndrome, small fiber polyneuropathy, pain, therapeutics, immunotherapy

## Abstract

Interstitial cystitis/bladder pain syndrome (IC/BPS) is a debilitating chronic disease that, based on the variable efficacy observed with most therapeutic options, is difficult to treat effectively. A more targeted patient selection process for current and emerging therapeutic options would likely help to improve outcomes. This narrative review explores small fiber polyneuropathy (SFPN) in IC/BPS as part of a larger widespread pain phenotype and as a potential therapeutic target. Because SFPN is becoming increasingly implicated in polysyndromic pain disorders (e.g., IC/BPS, chronic pelvic pain, and fibromyalgia) in which immune dysregulation is a suspected pathophysiologic etiology, continued consideration should be given to immunomodulatory therapies such as intravenous immunoglobulin (IVIg). Moreover, since the small fibers affected in SFPN continue to grow even as people age, targeted treatment may prevent further destruction and provide long-term benefits as the fibers are given time to repair. In addition to therapeutic potential, having a definitive SFPN diagnosis may provide psychological benefit in a patient population for which symptoms have historically been attributed to negative psychological factors. Finally, based on emerging data in this area, we propose consideration be given to include SFPN testing in the work-up of patients with IC/BPS that are refractory to treatments or have multiple comorbid pain syndromes since it may be an indicator of the need for alternative therapies. We believe that SFPN will play an increasingly larger role in the clinical evaluation and management of polysyndromic pain disorders, including IC/BPS.

## Introduction

Interstitial cystitis/bladder pain syndrome (IC/BPS) is a chronic condition characterized by pelvic pain perceived to be related to the bladder and concomitant voiding dysfunction. The heterogeneous nature of the presenting urological symptoms, together with numerous co-occurring non-urological associated syndromes (NUAS) and symptoms, poses a significant clinical challenge for diagnosis and treatment. Although many therapeutic options are available for patients with IC/BPS, there are currently no specific treatments that are effective in most patients and as a result, a diminished quality of life (QoL) is common. To promote the best outcomes for patients, it is necessary for clinicians to perform a thorough and holistic evaluation of each patient and practice shared decision making to devise a therapeutic strategy that is tailored to the needs of that individual (1).

One practical approach for improving therapeutic outcomes among IC/BPS patients is to stratify the heterogeneous group of patients into subgroups that share common characteristics. It is believed that utilizing therapeutic agents to target a specific subgroup’s unique feature(s), rather than the entire patient population as a whole, would exceed the response and outcome measures of the larger IC/BPS patient pool. With this concept as the guiding principle, we have characterized and described what we believe are two primary phenotypic subgroups within IC/BPS. The first group, representing 85-90% of all IC/BPS patients and which we refer to as *non-bladder centric*, is characterized by a non-low (> 500 cc) anesthetic bladder capacity (BC) and, on average, more co-occurring NUAS and symptoms. The second group, which we refer to as *bladder-centric*, comprises the other 10-15% of patients with IC/BPS and are typically older, have higher symptom scores on validated questionnaires, are more likely to have Hunner lesions, and have, by our definition, a low (≤ 500 cc) anesthetic BC (2–4). This group also includes end-stage bladder patients who are most likely to undergo major surgery for their IC/BPS such as cystectomy with urinary diversion.

Although IC/BPS patients within both phenotypic subgroups report having other co-occurring systemic pain disorders such as irritable bowel syndrome (IBS), urologic chronic pelvic pain syndrome (UCPPS), and fibromyalgia (FM), the bladder-centric patients tend to experience, on average, significantly fewer of these concomitant comorbid conditions. Therefore, we have begun to think of bladder-centric IC/BPS as a progressive disease of the bladder whereas non-bladder centric IC/BPS may represent a systemic (i.e., widespread beyond the bladder) pain disorder (5).

One emerging theme within this ‘systemic pain disorder’ concept in IC/BPS is the presence of small fiber polyneuropathy (SFPN). SFPN has been reported as a common finding in FM and UCPPS, and we have recently reported that approximately one-third of patients with IC/BPS have a confirmed finding of SFPN independent of diabetes mellitus (DM) (6). In this narrative review we describe SFPN and its presumed role in several chronic pain disorders that are directly related to IC/BPS. The primary question we intend to explore is: If a provider can confirm that a patient with IC/BPS has SFPN, in what way does that inform and change clinical management?

## Small fiber polyneuropathy

Peripheral neuropathies refer to disorders which involve nerves outside of the central nervous system, including ganglia, nerve trunks, and the autonomic nervous system. Further classifications can be made based on the focality and mechanism of damage to the fiber. In many cases the resulting degradation and damage evident in these fibers is the result of a systemic disease such as diabetes mellitus (DM), alcoholism, or infections, however many cases are idiopathic. Once affected, patients may commonly experience paresthesia, pain, weakness, and other autonomic symptoms. SFPN, a subgroup of peripheral neuropathy, is a peripheral nerve disorder affecting three fibers: thin myelinated Aδ, unmyelinated C, and autonomic axons. Neuropathy in these fibers generally results in altered mediation of temperature, pain, and autonomic function, respectively (7, 8). These symptoms may appear in a symmetrical length-dependent (LD) pattern, classic “stocking-glove” pattern such as in DM, or in a non-length dependent (NLD) pattern, appearing widely spread across the body (9). An interesting feature of these small fibers is that they continue to grow late into life, suggesting that an appropriate targeted treatment may be able to prevent further destruction and allow for regrowth of damaged fibers resulting in long-term benefits (10).

Various methods have been developed to evaluate small nerve fiber status, including questionnaires and autonomic nerve testing; however, the most direct approach to confirm SFPN is *via* a 3 mm skin punch biopsy from the right lower leg above the medial malleolus (11). Since the majority of these fibers are located in the epidermis, immunostaining for protein gene product (PGP) 9.5 can be utilized to visualize and quantify the intraepithelial nerve C fibers (IENF). A decrease in the intraepithelial nerve fiber density (IENFD), determined by agreed-upon counting rules and comparison to normative reference values, is diagnostic for SFPN (11). Following the original implementation of this methodology for quantitatively determining IENFD in the 1990s, associations of SFPN with various pain disorders have subsequently been reported. For example, Oaklander reported that ~50% of patients with FM are SFPN positive (12).

## SFPN in fibromyalgia

FM is a neurosensory condition characterized by chronic, widespread pain and an increase in musculoskeletal sensitivity to pressure. The exact etiology remains unclear, but it is thought to involve an interplay between peripheral nerve alterations and a form of central sensitization syndrome (13, 14). This term refers to the idea that the central nervous system plays a role in the amplification of pain, and possibly the origin of at least part of the pain, experienced by these individuals. However, it does not discount that abnormal nociceptive input from the periphery still plays a significant role in these individuals’ pain (13). Often, FM is a comorbid diagnosis in patients with similar chronic pain syndromes like IC/BPS and UCPPS (14). More specifically, patients in the non-bladder centric phenotype in IC/BPS are thought to have more systemic non-urologic symptoms and have been shown in preliminary studies to contain more SFPN+ individuals (5, 6). Given the prevalence of SFPN in these co-occurring conditions, it is possible that SFPN may also contribute to the pain aspects of IC/BPS. Therefore, it is reasonable to examine the implications of SFPN in FM and other similar/overlapping pain syndromes to determine the possible role that SFPN might play as a therapeutic target in the management of patients with IC/BPS.

## SFPN in CPP

Complex chronic pelvic pain (CPP) is another chronic pain syndrome that exhibits significant overlap with IC/BPS in terms of symptoms and associated conditions. In a 2019 case series of patients with complex CPP, it was found that 25/39 (64%) patients were found to be SFPN+. Interestingly, 38% of patients also had FM and 18% had IC/BPS (15). Importantly, Oaklander et al. also reemphasized the critical point that there may be a psychological benefit to identifying SFPN in patients with a disease that has historically been solely attributed to negative psychological factors. The prior findings attributing the majority of these patients’ symptoms to psychological factors can be quite disheartening to this suffering portion of the population. Moreso, this lack of validation could be an additional source of declining mental health for these patients. As mentioned previously, a growing body of evidence suggests that SFPN plays a role in many of these pain disorders, indicating that an increasing number of patients could experience psychological benefit from this more tangible rationale for their symptoms.

## General treatments for SFPN

Although there are not yet specific guidelines for the treatment of SFPN, the leading recommendations are centered on treatment of general neuropathic pain (8). To date, drugs such as serotonin-norepinephrine reuptake inhibitors (SNRIs), tricyclic antidepressants (TCAs), and anticonvulsants have shown the most efficacy (8). Outside of these general treatment recommendations there are no specific treatment options for SFPN, however there is evidence to suggest that some forms of SFPN may be amenable to an immunotherapeutic approach when autoimmune involvement is suspected. Liu et al. argues that the second largest group of SFPN patients, “initially idiopathic,” often have a significant autoimmune and/or inflammatory component (10). In light of this, they advocated for further assessment of immunotherapies for these cases of “apparently autoimmune SFPN” (10).

## Autoimmune theory of IC/BPS

The autoimmune theory is supported in part by the idea that there is noticeable epidemiological overlap between patients with IC/BPS and other individuals with known autoimmune disease (16, 17). Additional support was gained from the finding that in IC/BPS the bladder wall is infiltrated with increased inflammatory cells, such as mast cells, T lymphocytes, and eosinophils (18, 19). Dysregulation of these cells promotes activation and proliferation resulting in increased secretion of cytokines which can contribute to the ongoing immune response (20–22). The continuous nature of this process over time is thought to contribute to neuronal sensitization and alterations in transmission of pain signals (20, 21). Furthermore, there are reports of serologic findings to suggest autoimmunity plays a role in the pathogenesis of IC/BPS. Common autoantibodies have been found in IC/BPS that are also implicated in atopic dermatitis and Sjögren’s syndrome (SS) (17, 23). Additionally, IgG autoantibodies targeting epithelium and muscle fibers have been found in IC/BPS (24, 25). In contrast to these supporting findings, there are studies that argue that the immune dysregulation is a consequence of tissue damage rather than the origin of the cascade (26, 27). In summary, autoimmunity remains a controversial theory for IC/BPS, yet there remains a lack of conclusive evidence to refute this as an etiology.

## Immunotherapies for SFPN

In a 2013 case series, Oaklander et al. reported that of 15 SFPN+ patients who were given immune modulatory therapy with corticosteroids, 10/15 (67%) improved and the addition of intravenous immunoglobulin (IVIg) allowed for improvement in 12/15 (80%) patients (28).

In a 2018 case series, Liu et al. examined the use of IVIg in 55 patients with what they termed “apparent autoimmune SFPN.” In this cohort, only 25% were determined to have systemic autoimmune diagnoses, while 75% were deemed to have “restricted” small-fiber autoimmunity. Additionally, 80% of the patients had abnormal blood tests suggesting the presence of dysimmunity. They reported that 77% of patients were ‘IVIg responders’, based on significant improvement in both primary outcomes, pain severity and autonomic function testing (10). This study suggests that there is a role for IVIg in specific patients with *apparently autoimmune* SFPN.

Conversely, the first double-blind randomized controlled trial evaluating the use of IVIg in patients with idiopathic SFPN concluded that IVIg had no significant effect on pain in patients with painful idiopathic SFPN (29). However, this study did exclude patients with a prominent NLD pattern, which arguably represents a significant portion of SFPN that appears in chronic pain syndromes as described previously.

Therefore, this does not necessarily detract from the possibility that IVIG could be efficacious in IC/BPS patients that are SFPN positive. Alternatively, IC/BPS patients exhibiting a pattern of LD-SFPN most likely shouldn’t be recommended IVIG therapy in their course of treatment at this time based on the results of this trial. Future randomized controlled trials including NLD-SFPN would be needed to corroborate this idea.

In a case series, Pindi Sala et al. examined patients with SS and SFPN refractory to conventional treatments (30). Although this study didn’t differentiate between LD and NLD-SFPN, they made the case that IVIG had long-term effects and clinicians should consider low doses for longer periods when treating SFPN patients. They reported 9/12 cases with no relapses at a mean follow-up of 25 months with significant improvements in metrics such as pain and quality of life. If IVIG proves to be efficacious and have long-lasting effects in IC/BPS with SFPN, then this would warrant investigation into the inclusion of long-term low dose IVIG into the treatment regimen.

While the unique role of IVIg in each disease for which it is used therapeutically is not fully understood, there are commonalities that apply universally to many conditions. Briefly, IVIg has been shown to neutralize auto-reactive T-cells, down regulate B-cell activity and native antibody production, restore homeostasis between pro-inflammatory and anti-inflammatory drivers, and dissuade infiltration of autoimmune cells across the blood-nerve barrier (31). Given this information, its role in the treatment of many neuromuscular and immune neuropathies seems logical. This thought process could be applied to SFPN and IC/BPS in which some theories implicate T-cells and a dysregulated immune response as a having a role in the disease. There is some evidence to suggest that in this setting, IVIg may be more efficacious in individuals who possess blood-based markers of dysimmunity/inflammation, such as antinuclear antibodies, elevated erythrocyte sedimentation rate, low complement component 4, low complement component 3, and Sjögren’s autoantibodies (10). As previously mentioned, similar blood-based markers of dysimmunity/inflammation have been found in patients with IC/BPS.

## Immunotherapies in FM

Prior work has reported that a subset of FM patients with findings suggestive of chronic inflammatory demyelinating polyneuropathy appeared to respond to IVIg. In the 15 patients treated with IVIg, there was significant improvement in pain (p = 0.05), tenderness (p = 0.01), and strength (p = 0.04) (32).

In a 2002 prospective cohort, Goebel et al. assessed the efficacy of IVIg in treating a host of chronic pain syndromes including FM which had the largest presence in the study (48/130 patients). There were 25/48 patients that experienced >25% pain relief, while 8 of these experienced >70% pain relief (33). Pain relief was measured as average pain intensity (API) in standardized diaries. None of the patients had been previously diagnosed with an autoimmune disease wherein IVIg had already proved efficacious. Results of this study should be interpreted with caution considering the lack of a control group and blinding, factors which can increase the likelihood for some placebo effect. Of note, at the time of this study, the presence of SFPN in patients with FM was not established. However, in applying more recent findings one could speculate that some portion of the patients involved in this study also had SFPN.

## Immunotherapies in IC/BPS

**Figure 1:**
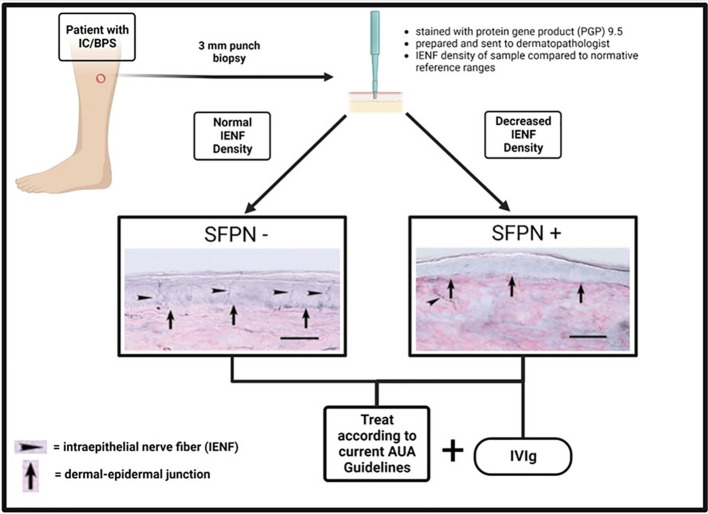
Schematic outlining the process for determination of SFPN status in IC/BPS patients and the corresponding treatment recommendations. (Created with BioRender.com).

Considering the overlap between IC/BPS and similar polysyndromic pain disorders that often seem to have an autoimmune component, it becomes sensible to consider further exploration of immunotherapies in patients with IC/BPS who are SFPN+ (**Figure 1**). Some attempts have already been made to utilize immunotherapies in IC/BPS regardless of presence of SFPN+. In 2022, Mykoniatis et al. reviewed the role of immunotherapies in IC/BPS including anti-tumor necrosis factor-α (TNF- α), adalimumab, certolizumab pegol, anti-nerve growth factors agents, tanezumab, and fulranumab (34). They concluded that only anti-TNF- α showed positive results in terms of superiority versus placebo in the treatment of IC/BPS, though this was based on a single study with encouraging results. Additional randomized controlled trials need to be conducted to better assess its efficacy. A 2020 Cochrane review of treatments for IC/BPS labeled immune modulators as being effective versus control, although the level of certainty was “very low” due to a small number of studies (35). However, the studies included did not assess the efficacy of immunomodulating treatments in subgroups of IC/BPS with SFPN, in which we would expect there to be a different, possibly improved, response to these treatments. The idea of a SFPN-targeted role for immunomodulating therapies has emerged in the aforementioned pain syndromes. The schematic in **Figure 1** outlines the decision tree by which SFPN testing could be implemented into the management of a patient with IC/BPS. Consideration of emerging therapeutics such as IVIg are not meant to replace the current AUA recommended treatments for IC/BPS; however, as additional controlled studies are performed, immunotherapy may prove to be a promising treatment option for this subset of patients.

## Conclusion

This review addressed some of the most relevant clinical implications of the complex relationship that is emerging between SFPN and chronic pain disorders including IC/BPS. The need to further elucidate this relationship is evident and routine SFPN testing should be included in the clinical work-up of patients with IC/BPS who are refractory to multiple treatments or have multiple comorbid pain syndromes. This new finding of SFPN in IC/BPS warrants contemplation when considering management strategies. A dysregulated immune system has been implicated in IC/BPS, in addition to other polysyndromic pain disorders, and has yet to be refuted as a leading theory. This provides momentum for the continued consideration of immunomodulatory therapies in SFPN. There is some evidence, albeit limited, that suggests dysimmune SFPNs may respond to immunomodulatory therapies including IVIg. Immunotherapies in this field require more randomized controlled trials before they should be accepted. SFPN is becoming increasingly implicated in polysyndromic pain disorders, which were in large previously attributed to negative psychological factors. Additionally, it is the authors’ opinion that there could be a psychological benefit for patients to learn of their underlying SFPN + diagnosis. In summary, we think that SFPN will play an increasingly larger role in how clinicians approach the treatment of polysyndromic pain in IC/BPS.

## Author contributions

SW conceived the topic and WW performed the literature search and initial drafting of the manuscript. SW, WW, MS, and PL edited the subsequent drafts. All authors contributed to the article and approved the submitted version.
